# Histone deacetylase 3 promotes alveolar epithelial–mesenchymal transition and fibroblast migration under hypoxic conditions

**DOI:** 10.1038/s12276-022-00796-y

**Published:** 2022-07-08

**Authors:** Sung Hwan Jeong, Eun Suk Son, Young Eun Lee, Sun Young Kyung, Jeong-Woong Park, Se-Hee Kim

**Affiliations:** 1 Department of Allergy, Pulmonary and Critical Care Medicine, Gachon University Gil Medical Center, Incheon, South Korea; 2 Department of Medicine, College of Medicine, Gachon University, Incheon, South Korea; 3 Gachon Medical Research Institute, Gachon University Gil Medical Center, Incheon, South Korea

**Keywords:** Mechanisms of disease, Epithelial-mesenchymal transition

## Abstract

Epithelial–mesenchymal transition (EMT), a process by which epithelial cells undergo a phenotypic conversion that leads to myofibroblast formation, plays a crucial role in the progression of idiopathic pulmonary fibrosis (IPF). Recently, it was revealed that hypoxia promotes alveolar EMT and that histone deacetylases (HDACs) are abnormally overexpressed in the lung tissues of IPF patients. In this study, we showed that HDAC3 regulated alveolar EMT markers via the AKT pathway during hypoxia and that inhibition of *HDAC3* expression by small interfering RNA (siRNA) decreased the migration ability and invasiveness of diseased human lung fibroblasts. Furthermore, we found that HDAC3 enhanced the migratory and invasive properties of fibroblasts by positively affecting the EMT process, which in turn was affected by the increased and decreased levels of microRNA (miR)-224 and Forkhead Box A1 (FOXA1), respectively. Lastly, we found this mechanism to be valid in an in vivo system; *HDAC3* siRNA administration inhibited bleomycin-induced pulmonary fibrosis in mice. Thus, it is reasonable to suggest that HDAC3 may accelerate pulmonary fibrosis progression under hypoxic conditions by enhancing EMT in alveolar cells through the regulation of miR-224 and FOXA1. This entire process, we believe, offers a novel therapeutic approach for pulmonary fibrosis.

## Lung fibrosis: Enzyme inhibition could limit disease progression

Inhibiting an enzyme that boosts the invasiveness of fibrosis-related cells could prove to be a novel therapeutic strategy for treating idiopathic lung fibrosis. Lung fibrosis progresses via the transition of epithelial cells into myofibroblasts, which are migratory invasive cell types that secrete collagen and deposit excessive extracellular material. Low oxygen conditions (hypoxia) accelerate this transition process. Scientists recently identified a group of histone deacetylases (HDACs) that are significantly overexpressed in the lung tissues of patients with fibrosis. In experiments on mice and human cell lines, Jeong-Woong Park and Se-Hee Kim at Gachon University Gil Medical Center, Incheon, South Korea, and co-workers demonstrated that under hypoxic conditions, HDAC3 increases the cellular transition to myofibroblasts by regulating the expression of a key microRNA and its target gene. Inhibiting HDAC3 suppresses the migration and invasiveness of lung myofibroblasts.

## Introduction

Epithelial–mesenchymal transition (EMT) is a cellular event that causes the conversion of epithelial cells into mesenchymal (fibroblast-like) cell types. Epithelial cells maintain cell–cell interactions through tight junctions, adherens junctions, and desmosomes. Conversely, mesenchymal cells acquire migratory and invasive properties through loss of cell–cell junctions and cell polarity^1^. EMT not only occurs during embryonic development but also is an important process in cancer metastasis and organ degeneration, such as that occurring through fibrosis^1^. Specifically, in fibrotic tissues, myofibroblasts secrete an excessive amount of collagen and deposit abundant extracellular matrix (ECM). A significant portion of these myofibroblasts arises from the conversion of epithelial cells via EMT^2,3^. However, the molecular mechanisms driving the progression of fibrosis via EMT, which plays an important role in the pathogenesis of idiopathic pulmonary fibrosis (IPF), are not fully understood.

IPF is a progressive and irreversible disease characterized by an abnormal fibrotic response and leading to death from respiratory failure^4^. Since the mechanism underlying IPF onset and progression is unclear, research on the molecular mechanism is crucial for the development of effective therapies, which are lacking.

Lung tissues are sensitive to hypoxia, which is further associated with inflammation and fibrosis in lung tissues and is the key pathological feature of pulmonary fibrosis^5–7^. Zhou et al. demonstrated that hypoxia induces alveolar EMT through mitochondrial reactive oxygen species and hypoxia-inducible factor-1 (HIF-1)^8^. Furthermore, Higgins et al. revealed that hypoxia promotes fibrogenesis via HIF-1-mediated induction of EMT^9^.

Histone deacetylases (HDACs) are enzymes that remove acetyl groups from the lysine residues of proteins. HDACs not only regulate chromatin structure and transcription through epigenetic modification of histones but also modulate diverse cellular processes via deacetylation of nonhistone proteins. HDACs are classified into four groups: class I HDACs (HDAC1, HDAC2, HDAC3, and HDAC8) are localized in the nucleus; class II HDACs (HDAC4, HDAC5, HDAC6, HDAC7, HDAC9, and HDAC10) are localized in the cytoplasm but shuttle to the nucleus; class III HDACs are sirtuins; and class IV contains only HDAC11. Class I, II, and IV HDACs are Zn^2+^-dependent enzymes, while class III HDACs are NAD^+^-dependent enzymes^10^. Importantly, HDAC inhibitors are considered potent anticancer drugs and show promise as therapeutic agents for many diseases, including pulmonary fibrosis^11^. Although HDAC inhibitors have been studied as therapeutic agents for pulmonary fibrosis, the functions of HDACs in pulmonary fibrosis have not been characterized.

MicroRNAs (miRNAs) are small noncoding RNAs that function as posttranscriptional regulators. They bind to their target messenger RNAs (mRNAs), which results in posttranscriptional gene silencing^12^. Each miRNA is able to regulate multiple genes, and each mRNA may be regulated by numerous miRNAs. Thus, miRNAs are involved in important biological functions. Recently, studies have reported that miRNAs regulate pulmonary fibrosis via upregulation of profibrotic miRNAs and downregulation of antifibrotic miRNAs, which regulate EMT in epithelial cells. Among miRNAs, miR-224 has been reported to regulate the migration, invasion, and apoptosis of hepatocellular carcinoma cells and to drive EMT via E2F1 to control cancer progression^13–15^. In this study, we aimed to identify the miRNAs that are controlled by HDAC3 under hypoxic conditions and to identify their target genes. In doing so, we intended to elucidate the mechanism by which HDAC3 accelerates pulmonary fibrosis progression.

## Materials and methods

### Cell lines and hypoxic conditions

The human alveolar epithelial cell line A549 and diseased human lung fibroblasts from patients with idiopathic pulmonary fibrosis (DHLF-IPF cells) were obtained from the Korean Cell Line Bank (Seoul, South Korea) and Lonza (Basel, Switzerland), respectively. A549 cells were cultured in RPMI-1640 medium (Gibco Cell Culture, Carlsbad, CA, USA) containing 10% fetal bovine serum (FBS; Gibco) and 1% penicillin–streptomycin (Gibco), and DHLF-IPF cells were grown using an FGM-2 Fibroblast Growth Medium-2 BulletKit (Lonza). For hypoxic incubation, cells were incubated in a hypoxic incubator (New Brunswick Scientific, Edison, NJ, USA) with a humidified environment consisting of 1% O_2_, 5% CO_2_, and 94% N_2_.

### Generation of stable cell lines

For lentivirus production, 293T cells were transfected with human HDAC3, empty lentiviral vectors (Applied Biological Materials [ABM], Richmond, BC, Canada), and a third-generation packaging mix (ABM) according to the manufacturer’s protocols. After collection of virus-infected A549 cells, transduced cells were selected with puromycin (Sigma–Aldrich, St. Louis, MO, USA) for 2 weeks.

### RNA interference, miR-224 inhibitors, miR-224 mimics, and transfection

Cells were transfected with 50 nM siRNAs (Bioneer, Daejeon, South Korea) or 50 nM miR inhibitors (Bioneer) or 5 nM miR mimics (Bioneer) using Lipofectamine 2000 (Invitrogen, Carlsbad, CA, USA) according to the manufacturer’s protocols.

The sequences of the siRNAs, miR-224 inhibitors, and miR-224 mimics were as follows: *HDAC1* (5′-CU AAU GAG CUU CCA UAC AA-3′), *HDAC2* (5′-UC CGU AAU GUU GCU CGA UG-3′), *HDAC3* (5′-AAU CAG AAC UCA CGC CAG UAU-3′ and 5′- GAU GCU GAA CCA UGC ACC-3′), *HDAC8* (5′-UCA ACU ACA UCA AAG GGA AUC UGA A-3′), *HIF1A* (5′-AG UUC ACC UGA GCC UAA UA-3′ and 5′-GC ACA GUU ACA GUA UUC CA-3′), *TM4SF1* (Transmembrane 4 S Six Family Member 1) (5′-GCC UCU CAA UGU GGU UCC CUG GAA U-3′), *PRKAA1* (Protein Kinase AMP-Activated Catalytic Subunit Alpha 1) (5′-GCG UGU ACG AAG GAA GAA U-3′), *FOXA1* (Forkhead Box A1) (5′-GCG UGG AUU CGG UGU GGA AUC A-3′), *WT1* (Wilms tumor 1)(5′-AAA UAU CUC UUA UUG CAG CCU GGG U-3′), *PTGR1* (Prostaglandin Reductase 1) (5′-GCC AAA GUU GUG GAA AGU A), miR-224-3p inhibitor (5′-AAA AUG GUG CCC UAG UGA CUA CA-3′), and miR-224 mimic (5′-UCA AGU CAC UAG UGG UUC CGU UUA G-3′).

### Western blot analysis

For protein preparation, cells were lysed with radioimmunoprecipitation assay buffer (50 mM Tris-HCl (pH 8.0), 150 mM NaCl, 0.5% sodium deoxycholate, 0.1% sodium dodecyl sulfate, and 1% NP-40) or whole-cell lysis buffer (10 mM 4-(2-hydroxyethyl)-1-piperazineethanesultonic acid (pH 7.9), 400 mM NaCl, 0.1 mM ethylenediaminetetraacetic acid, 5% glycerol, and 1 mM dithiothreitol). Primary antibodies against Fibronectin (Santa Cruz Biotechnology, Santa Cruz, CA, USA), E-cadherin (Cell Signaling, Beverly, MA, USA), alpha smooth muscle actin (α-SMA) (Abcam, Cambridge, UK), Zinc finger E-box binding homeobox1 (ZEB1) (Cell Signaling), N-cadherin (Cell Signaling), Vimentin (Cell Signaling), β-catenin (Cell Signaling), HIF-1α (Novus, Centennial, CO, USA), Snail (Cell Signaling), HDAC1 (Santa Cruz Biotechnology), HDAC2 (Santa Cruz Biotechnology), HDAC3 (Santa Cruz Biotechnology), HDAC8 (GeneTex, Irvine, CA, USA), phospho-Ak strain transforming (AKT) (Cell Signaling), AKT (Cell Signaling), phospho-Extracellular signal regulated kinase (ERK)1/2 (Cell Signaling), ERK1/2 (Cell Signaling), phospho-p65 (Cell Signaling), p65 (Cell Signaling), phospho-c-Jun N-terminal kinase (JNK) (Cell Signaling), JNK (Cell Signaling), phospho-Small mothers against decapentaplegic (SMAD)2/3 (Cell Signaling), SMAD2/3 (Cell Signaling), phospho-Signal transducer and activator of transcription (STAT) 3 (Cell Signaling), STAT3 (Cell Signaling), Forkhead Box A1 (FOXA1) (Abcam), and β-actin (Santa Cruz Biotechnology) were used. Expression was normalized to β-actin or quantified as a ratio of phosphorylated protein to total protein using ImageJ software (NIH, Bethesda, ML, USA).

### Next-generation sequencing (NGS) analysis for miRNA and mRNA expression profiling

Samples were prepared from stable A549-Blank (BLK) and A549-HDAC3 cells, and total RNA was extracted using TRIzol reagent (Thermo Fisher Scientific, Waltham, MA, USA). All procedures for NGS analysis were performed by Macrogen (Seoul, South Korea). Predicted human miRNA–mRNA interaction databases were generated with TargetScan (QIAGEN, Germantown, MD, USA). TargetScan predicts miRNA–mRNA interactions by searching for conserved 6-8-mer binding sites in mRNAs, matching these sites with miRNA seed sequences and taking into account the surrounding sequences.

### Quantification of mature miR-224 expression

Total RNA was prepared from cells using the miRNeasy Kit (QIAGEN) and was reverse transcribed using a MicroRNA First-Strand Synthesis Kit (Takara Bio, Otsu, Japan). Quantitative real-time PCR was performed on miRNA using SYBR Green I Universal PCR Master Mix (Takara Bio) and a probe specific for miR-224 on a CFX96 Real-Time PCR Detection System (Bio–Rad, Hercules, CA, USA). U6 snRNA was used as an endogenous control. The primer sequences for miR-224 amplification were as follows: human-miR-224 (5′-GTG GTT CCG TTT AGT AGA TGA TTG TGC ATT G-3′) and mouse-miR-224-3p (5′-AAA TGG TGC CCT AGT GAC TAC A-3′). Samples were loaded in triplicate, and the experiment was repeated more than three times.

### Quantitative reverse transcription PCR (qRT–PCR)

Total RNA was prepared from cells using RNAiso Plus reagent (Takara Bio), and a PrimeScript First Strand cDNA Synthesis Kit (Takara Bio) was employed for cDNA transcription. qRT–PCR was performed using SYBR Green I Universal PCR Master Mix (Takara Bio) on a CFX96 real-time system (Bio–Rad). Samples were loaded in triplicate, and the experiment was repeated more than three times. The fold change in target gene expression relative to the endogenous control gene *GAPDH* (Glyceraldehyde 3-phosphate dehydrogenase) was determined following the manufacturer’s instructions. The primer sequences used for PCRs were as follows: human *CDH1* (Cadherin 1), 5′- CTG AGA ACG AGG CTA ACG-3′ (forward) and 5′- GTC CAC CAT CAT CAT TCA ATA T-3′ (reverse); human *SLUG*, 5′-ACG CCC AGC TAC CCA ATG-3′ (forward) and 5′-CGC CCC AAA GAT GAG GAG TA-3′ (reverse); human *SNAI1*, 5′-CCC CAA TCG GAA GCC TAA CT-3′ (forward) and 5′-GCT GGA AGG TAA ACT CTG GAT TAG A-3′ (reverse); human *TWIST1*, 5′-GCG CTG CGG AAG ATC ATC-3′ (forward) and 5′-GGT CTG AAT CTT GCT CAG CTT GT-3′ (reverse); human *VEGFA* (Vascular Endothelial Growth Factor A), 5′-ATC TTC AAG CCA TCC TGT GTG C-3′ (forward) and 5′-CAA GGC CCA CAG GGA TTT TC-3′ (reverse); human *HDAC3*, 5′- TAG ACA AGG ACT GAG ATT GCC-3′ (forward) and 5′-GTG TTA GGG AGC CAG AGC C-3′ (reverse); human *FOXA1*, 5′-GCA ATA CTC GCC TTA CGG CT-3′ (forward) and 5′-TAC ACA CCT TGG TAG TAC GCC-3′ (reverse); human *GAPDH*, 5′-AGG TCG GAG TCA ACG GAT TTG G-3′ (forward) and 5′-ACA GTC TTC TGG GTG GCA GTG ATG-3′ (reverse); mouse *Foxa1*, 5′-GCC TTA CTC CTA CAT CTC GCT C-3′ (forward) and 5′-CTG CTG GTT CTG GCG GTA ATA G-3′ (reverse); and mouse *Gapdh*, 5′-TGT GTC CGT CGT GGA TCT GA-3′ (forward) and 5′-CCT GCT TCA CCA CCT TCT TGA-3′ (reverse).

### Immunocytochemistry

Cells were seeded on coverslips coated with 0.1% gelatin in 6-well plates, incubated under hypoxic/normoxic conditions for 4 days, and then fixed with 3.7% paraformaldehyde (Junsei Chemical, Tokyo, Japan). After permeabilization in 0.5% Triton X-100 (VWR Life Science, Radnor, PA, USA)/phosphate-buffered saline (PBS-T) and several washes with PBS, the cells were incubated with an anti-E-cadherin antibody (Cell Signaling) diluted in 1% bovine serum albumin (BSA)(MP Biomedicals, Santa Ana, CA, USA)/PBS-T for 1 h at room temperature. Afterward, the cells were incubated with Alexa Fluor 555 donkey anti-rabbit IgG (Life Technologies, Carlsbad, CA, USA) and mounted in Vectashield containing DAPI (Vector Laboratories, Burlingame, CA, USA). Images were acquired at 200× magnification with an LSM710 confocal microscope (Carl Zeiss, Jena, Germany).

### Luciferase reporter assay

Stable A549-BLK and A549-HDAC3 cells were transfected with the pSV40 promoter-EpoHRE-Luciferase reporter and pCMV-β-galactosidase (gal) plasmids using Lipofectamine 2000 (Invitrogen). The cells were then incubated under normoxic or hypoxic conditions for 24 h. Luciferase reporter assays (Promega, Madison, WI, USA) were conducted using the same amount of cell lysates and corrected for transfection efficiency using a β-gal assay (Promega). The luciferase reporter and β-gal assays were performed following the manufacturer’s instructions.

To validate the relationship between miR-224 and its target gene FOXA1, miR-224 mimic/inhibitor and pEZX-luc-FOXA1 3′-UTR vector (GeneCopoeia, Rockville, MD, USA) were transfected into A549 cells using RNAiMAX (Invitrogen). After the cells were incubated for 72 h, the levels of the secreted reporter proteins GLuc and SEAP were measured using the supernatant following the manufacturer’s instructions.

### Wound healing assay

Cells were seeded in 60-mm dishes, and the cell layer was then scratched with a pipette tip after 24 h of incubation. Images were acquired at 0 and 24 h using an Olympus CFX41 microscope (Hamburg, Germany). The cell migration area was quantified using ImageJ software and analyzed as described previously^16^.

### Transwell migration assay

The experimental process was described previously^17^. Briefly, siRNA-transfected cells were seeded into Transwell chambers with the lower surface of the membrane coated with 0.2% gelatin, and medium containing 20% FBS was used as a chemoattractant. After 24 h of incubation under normoxic or hypoxic conditions, the cells on the lower surface of the membrane were fixed and stained using crystal violet (YD Diagnostics, Gyeonggi, South Korea) for counting. The experiments were independently performed in triplicate.

### Matrigel invasion assay

This assay was performed as previously described^17^. Briefly, siRNA-transfected cells were seeded into Transwell chambers with the lower surface of the membrane coated with 0.2% gelatin and the upper surface coated with Matrigel (BD Biosciences, San Jose, CA, USA). After 48 h of incubation under normoxic or hypoxic conditions, the cells on the lower surface of the membrane were fixed and stained using crystal violet (YD Diagnostics) for counting. The experiments were independently performed in triplicate.

### Mouse model of lung fibrosis and analysis of fibrosis in vivo

We purchased male C57BL/6 N mice, weighing an average of 20–25 g at the age of 6–7 weeks (Japan SLC, Shizuoka, Japan) and subjected these mice to intratracheal instillation of bleomycin (3.5 units/kg) (Sigma–Aldrich). Three micrograms of siRNA for in vivo use (Accell SMARTpool siRNA) (Dharmacon, Lafayette, CO, USA) diluted in 60 μL Accell Delivery Media (Dharmacon) was administered intratracheally on Days 2, 5, and 12 after bleomycin treatment. All mice were anesthetized and sacrificed on Day 22, and lung samples were weighed and collected. The left lung samples were fixed overnight with 10% formalin and embedded in paraffin. The samples were stained with hematoxylin (Merck, Darmstadt, Germany) and eosin (Sigma–Aldrich) to investigate histological changes and were stained with Masson’s trichrome stain (Sigma–Aldrich) to evaluate collagen deposition within the lung parenchyma. At least three sections from each mouse were randomly selected, and 20 fields per section were used for immunohistochemical evaluation. The right lungs were minced into small pieces and used for protein and RNA extraction and collagen content analysis.

The amount of collagen in lung tissues was determined by measuring the content of hydroxyproline using a Hydroxyproline assay kit (Abcam, Cabridge, UK). Briefly, lung tissues were extracted and homogenized. After hydrolyzing and neutralizing the homogenate, the supernatant was collected and the amount of hydroxyproline was measured using the supernatant following the manufacturer’s instructions.

### Immunohistochemistry

The expression levels of Carbonic anhydrase IX (CAIX), HDAC3, Snail, and FOXA1 were evaluated by immunohistochemistry. Sections with a 6-μm thickness were prepared by slicing lung tissue blocks embedded in paraffin. Slides were deparaffinized in xylene and rehydrated through a series of alcohols in water. Antigen retrieval was performed by incubation in citrate buffer (pH 6) in a microwave for 20 min. Endogenous peroxidase activity was blocked by adding 3% H_2_O_2_ for 10 min. After the samples were incubated overnight with primary antibodies against CAIX (Novus), HDAC3 (GeneTex, Irvine, CA, USA), Snail (Sigma–Aldrich), and FOXA1 (Abcam) at 4 °C, the samples were rinsed and incubated with secondary antibodies. Diaminobenzidine (Roche, Germany) was added, and the slides were incubated in the dark for 10 min. Expression levels were quantified using ImageJ software.

### Patient samples

The study population consisted of paraffin-embedded lung tissue samples from five IPF patients and histologically defined normal lung sections from five control subjects who had undergone surgery for lung cancer. The IPF patient samples were reviewed by a pathologist to confirm the diagnosis of usual interstitial pneumonia.

### Statistics

Results were analyzed using GraphPad Prism (San Diego, CA, USA). Statistical significance was assessed using the Mann–Whitney test and two-tailed paired *t* test; *p* < 0.05 was considered significant.

### Study approval

Institutional and national guidelines for the care and use of mice were followed, and all experimental procedures involving mice were approved by the Institutional Animal Care and Use Committee (MRI-2019-0001). Research involving human subjects was approved by the Gil Medical Center Institutional Review Board (IRB GFIRB2019-421).

## Results

### HDAC3 regulates the expression of epithelial–mesenchymal markers via the AKT pathway during hypoxia

To test whether hypoxia affects alveolar EMT, we exposed the human and rat alveolar epithelial cell lines A549 and RLE-6TN, respectively, to hypoxia for 4–8 days. As shown in Supplementary Fig. 1a, b, hypoxia induced a 28–81% decrease in the protein level of the epithelial cell marker E-cadherin in both cell lines compared to the corresponding normoxic control cells. In addition, the expression level of the mesenchymal marker Fibronectin showed a 14–475% increase under hypoxic conditions. Next, we examined the mRNA levels of genes involved in EMT in A549 cells after exposure to hypoxia. As shown in Supplementary Fig. 1c, the mRNA levels of the mesenchymal markers *SNAI1* (107–238%), *SLUG* (110–305%), *TWIST1* (102–228%), *FN1* (Fibronectin1) (138–267%), *ACTA2* (actin alpha 2, smooth muscle) (111–191%), and *COL1A1* (collagen type I alpha 1 chain) (99–252%) were elevated under hypoxic conditions compared to normoxic control conditions. Vascular endothelial growth factor (VEGF) was used as a positive control for hypoxic conditions. Taken together, these results suggest that hypoxia induces alveolar EMT.

**Fig. 1:**
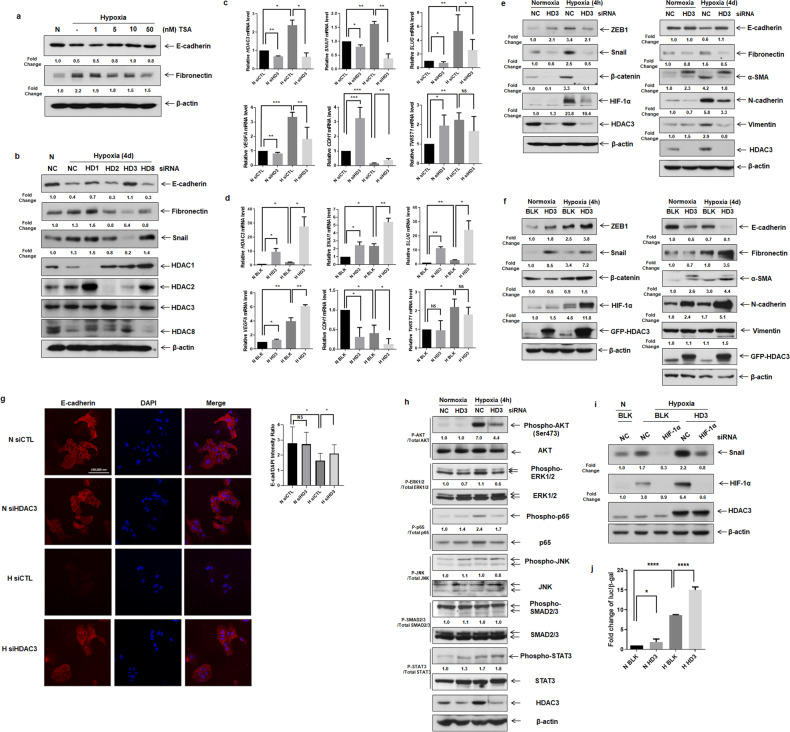
HDAC3 is an effective regulator of EMT markers via the AKT pathway under hypoxia. **a** Immunoblot analysis of A549 cells treated with TSA and exposed to normoxia/hypoxia for 4 days. **b** Immunoblot analysis of A549 cells transfected with siRNAs against *HDAC1, HDAC2, HDAC3*, and *HDAC8* and exposed to normoxia/hypoxia for 4 days. **c**, **d** qRT–PCR analysis of A549 cells overexpressing HDAC3 or A549 cells transfected with *HDAC3* siRNA and exposed to normoxia/hypoxia for 4 h. **e**, **f** Immunoblot analysis of A549-HDAC3 cells and A549 cells transfected with *HDAC3* siRNA and exposed to normoxia/hypoxia for 4 h or 4 days. **g** Representative immunocytochemical images of A549 cells transfected with *HDAC3* siRNA and exposed to normoxia/hypoxia for 4 days (left). Quantification of E-cadherin-positive cells (right). **h** Immunoblot analysis of A549 cells transfected with *HDAC3* siRNA and exposed to normoxia/hypoxia for 4 h. **i** Immunoblot analysis of stable A549-BLK and A549-HDAC3 cells transfected with siRNA against *HIF1A* and exposed to normoxia/hypoxia for 4 h. **j** Luciferase reporter assay of stable A549-BLK/HDAC3 cells transfected with pSV40pro-EpoHRE-Luc and pCMV-β-gal and incubated under normoxia/hypoxia for 24 h. N, normoxia; H, hypoxia; HD, histone deacetylase; NC, negative control; BLK, blank; TSA, trichostain A; AKT, ak strain transforming; ERK, extracellular signal regulated kinase; JNK, c-jun N-terminal kinase; SMAD, small-mothers-against-decapentaplegic; STAT, signal transducer and activator of transcription. *P < 0.05, **P < 0.01, ***P < 0.001, and ****P < 0.0001 vs. control. NS, not significant. Scale bar, 100 µm.

To examine whether HDACs regulate hypoxia-induced EMT, we treated A549 cells with an HDAC inhibitor, trichostatin A (TSA), and then exposed them to hypoxia for 4 days. As shown in Fig. 1a, TSA-treated A549 cells under hypoxia showed a 60–100% increase in the E-cadherin protein level compared to that in hypoxic A549 cells not treated with TSA, whereas the level of Fibronectin was reduced by 14–32% in hypoxic TSA-treated cells. Next, we sought to determine which HDAC classes affect hypoxia-induced alveolar EMT using siRNAs against *HDACs*. Among the class I HDACs, HDAC3 was effective in modulating E-cadherin, Fibronectin, and Snail expression under hypoxic conditions (Fig. 1b). These results demonstrate that HDAC3 regulates hypoxia-induced alveolar EMT. The expression level of HDAC3 was slightly elevated in hypoxic A549 and RLE-6TN cells (Supplementary Fig. 1a–c). To confirm the regulatory effect of HDAC3 on hypoxia-induced alveolar EMT, we transfected A549 cells with siRNA against *HDAC3* and then incubated the cells under hypoxic conditions. During hypoxia, inhibition of HDAC3 increased the *CDH1* (Cadherin 1) mRNA level (100–300%) and decreased the mRNA levels of *SNAI1* (70–88%) and *SLUG* (33–84%) compared to those in hypoxic control cells (Fig. 1c). To examine the effects of HDAC3 on the expression of EMT markers at the protein level, we performed Western blot analysis using lysates from cells transfected with siRNA against *HDAC3*. HDAC3-deficient A549 and RLE-6TN cells showed increases of 83% and 180%, respectively, in the protein expression level of E-cadherin under hypoxia, whereas the expression levels of ZEB1 (A549: 38%, RLE-6TN: 57%), Snail (80%, 75%), β-catenin (97%, 94%), Fibronectin (69%, 55%), α-SMA (57%, 92%), N-cadherin (43%, 45%), and Vimentin (72%, 57%) were decreased by inhibition of HDAC3 (Fig. 1e and Supplementary Fig. 1d). Furthermore, knockdown of HDAC3 suppressed HIF-1α expression in hypoxic alveolar epithelial cells. To verify the regulatory effect of HDAC3 on hypoxia-induced alveolar EMT, we established a cell line with overexpression of HDAC3 by lentiviral transduction of A549 cells. As shown in Fig. 1d, overexpression of HDAC3 decreased the mRNA level of *CDH1* (38–86%) and increased those of *SNAI1* (100–159%) and *SLUG* (617–890%) under hypoxia compared to the corresponding levels in hypoxic control cells. In addition, overexpression of HDAC3 reduced the E-cadherin protein level (86%), whereas HDAC3 overexpression increased the protein levels of ZEB1 (52%), Snail (112%), β-catenin (67%), Fibronectin (94%), α-SMA (47%), N-cadherin (200%), and Vimentin (36%) under hypoxia (Fig. 1f). Inhibition and overexpression of HDAC3 did not affect hypoxia-induced *TWIST1* mRNA expression (Fig. 1c, d). Thus, we posit that the regulatory effect of HDAC3 on hypoxia-induced EMT is dependent on Snail but not on Twist. To observe how the cellular distribution of E-cadherin expression is regulated by HDAC3 during hypoxia, we performed immunofluorescence staining in alveolar epithelial cells. In A549 and RLE-6TN cells transfected with *HDAC3* siRNA, E-cadherin staining was performed using an appropriate antibody, and E-cadherin was detected in cell–cell contacts. Consistent with previous results, E-cadherin expression was lost after exposure to hypoxia and was restored by inhibition of HDAC3 in hypoxic epithelial cells (Fig. 1g and Supplementary Fig. 1e). Taken together, these findings suggest that HDAC3 is effective in modulating alveolar EMT under hypoxic conditions.

To investigate the signaling pathways controlled by HDAC3 during hypoxia-induced EMT, we examined the AKT^18^, ERK1/2^19^, p65^14^, JNK^20^, SMAD2/3^21^, and STAT3^22^ pathways in HDAC3-deficient alveolar epithelial cells by Western blot analysis. As shown in Fig. 1h and Supplementary Fig. 1f, *HDAC3*-silenced A549 and RLE-6TN cells showed decreases of 37.1% and 62.2%, respectively, in hypoxia-induced AKT activation. In addition, the inhibitory effect of HDAC3 on the phosphorylation of ERK1/2 and p65 was less than that of AKT. Taken together, these findings suggest that HDAC3 modulates alveolar EMT via AKT activation under hypoxia.

We hypothesize that HDAC3 may trigger Snail-dependent EMT through recruitment of HIF-1α. Among the EMT markers, we focused on Snail because Snail, as a major repressor of E-cadherin, plays a pivotal role in the EMT process. To determine the correlations among HDAC3, HIF-1α, and Snail expression, siRNA against *HIF1A* was transfected into stable A549-HDAC3 cells. Inhibition of HIF-1α prevented the HDAC3-induced increase in Snail expression (Fig. 1i). Therefore, we suggest that HDAC3 modulates Snail expression through stabilization of HIF-1α under hypoxic conditions. In addition, to examine the mechanism by which HDAC3 enhances the transcriptional activity of HIF-1α during the EMT process, we performed a luciferase reporter assay. As shown in Fig. 1j, under hypoxic conditions, HDAC3 showed a 52.8–88.1% increase in the transcription of the hypoxia-response element (HRE) to which HIF-1α and HIF-1β bind. Therefore, these data suggest that HDAC3 stabilizes HIF-1α by increasing the transcription of the HRE bound by HIF-1α and that the stabilized HIF-1α protein may seem to induce the EMT process by increasing Snail expression.

### HDAC3 increases the migration and invasion of fibroblast under hypoxic conditions

**Fig. 2:**
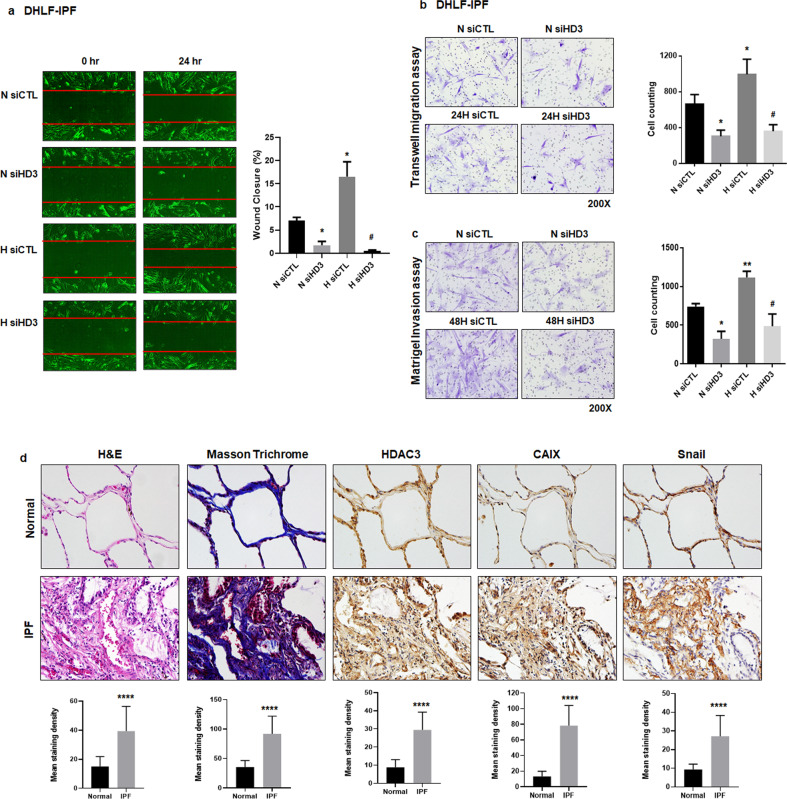
Inhibition of HDAC3 prevents the migration and invasion of fibroblast cells under hypoxic conditions. **a**–**c** DHLF-IPF cells were transfected with *HDAC3* siRNA and incubated under normoxia/hypoxia for 24–48 h. **a** Representative images showing the wound healing assay (left). Quantification of the percentage of healed area (right). Magnification, 40×. **b** Representative images showing migrated cells (left). Quantification of migrated cells (right). Magnification, 200×. **c** Representative images showing invaded cells (left). Quantification of invaded cells (right). Magnification, 200×. **d** Histological and immunohistochemical evaluation of lung tissues from IPF patients (*n* = 5). H&E (Hematoxylin/eosin) staining, Masson’s trichrome staining, and immunohistochemical staining for HDAC3, CAIX, and Snail. Representative images (top) and quantification (bottom) of staining are shown. Magnification, 400×. N, normoxia; H, hypoxia; CTL, control; HD, histone deacetylase; IPF, idiopathic pulmonary fibrosis; CAIX, carbonic anhydrase IX. *P < 0.05, **P < 0.01, and ****P < 0.0001 vs. normal or normoxic control; ^#^*P* < 0.05 vs. hypoxic control.

To test whether HDAC3 regulates the migration and invasion of human fibroblast cells under hypoxic conditions, we performed a scratch wound healing assay using diseased human lung fibroblasts from patients with idiopathic pulmonary fibrosis (DHLF-IPF cells) and MRC-5 human lung fibroblast cells. As shown in Fig. 2a and Supplementary Fig. 2a, under hypoxic conditions, *HDAC3* repression via siRNA decreased wound healing in DHLF-IPF (96.7–98.3%) and MRC-5 (85.5–95.8%) cells. To confirm the regulatory effect of HDAC3 on fibroblast migration, we conducted a Transwell migration assay. Consistent with the above findings, DHLF-IPF and MRC-5 cells showed decreases of 54.5–75.5% and 39.3–88.6%, respectively, in hypoxia-induced migration after inhibition of *HDAC3* using siRNA (Fig. 2b and Supplementary Fig. 2b). Recently, it has been reported that fibrotic fibroblast and myofibroblast cells promote fibrogenesis via invasion and degradation of basement membranes^23^. Therefore, to examine whether HDAC3 affects fibroblast invasiveness under hypoxia, we performed a Matrigel invasion assay. Hypoxia increased the invasiveness of fibroblast cells, whereas inhibition of *HDAC3* using siRNA caused decreases of 46.7–72.2% and 32.9–56.1% in hypoxia-induced invasion in DHLF-IPF and MRC-5 cells, respectively (Fig. 2c and Supplementary Fig. 2c). Taken together, these data suggest that HDAC3 enhances the migratory and invasive properties of human fibroblast cells under hypoxic conditions.

To evaluate whether HDAC3 expression is elevated in fibrotic lung tissues, we performed immunohistochemistry with lung tissues from human IPF patients. We observed increased lung injury by hematoxylin/eosin and Masson’s trichrome staining (Fig. 2d). Furthermore, the expression level of HDAC3 was increased by 188-290% in lung tissues from IPF patients (Fig. 2d). The Carbonic anhydrase IX (CAIX) gene is hypoxia inducible and a useful histochemical marker for hypoxia. As expected, CAIX and Snail showed increases of 325-747% and 127-223%, respectively, in lung tissues from human IPF patients (Fig. 2d). These results demonstrate that the expression levels of HDAC3, CAIX, and Snail are increased in pulmonary fibrosis tissues, suggesting that HDAC3, CAIX, and Snail may be involved in pulmonary fibrosis.

### siRNA-mediated silencing of *HDAC3* ameliorates lung fibrosis induced by bleomycin

**Fig. 3:**
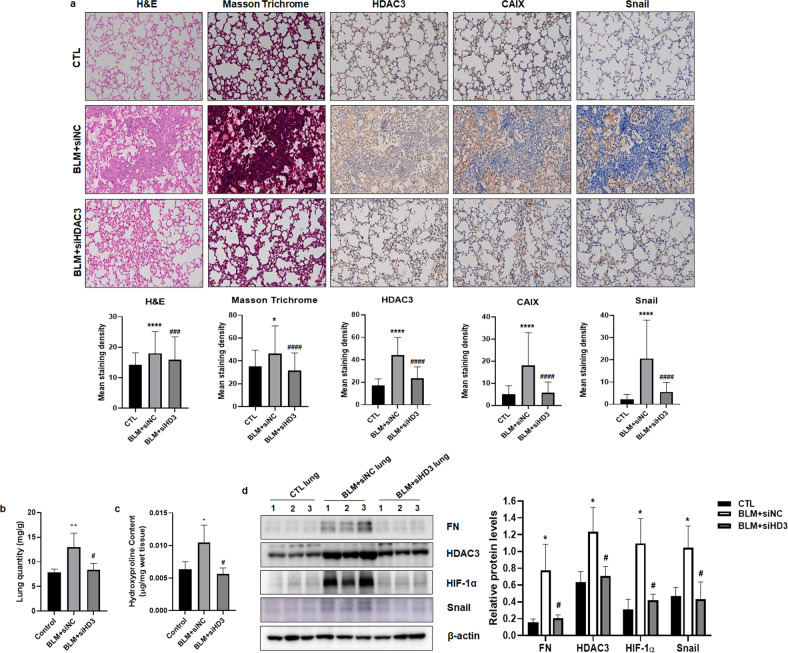
Inhibition of HDAC3 prevents bleomycin-induced pulmonary fibrosis in mice. **a** Histological and immunohistochemical evaluation of lung tissues from control mice and bleomycin-injected mice treated with negative control siRNA or *HDAC3* siRNA (*n* = 3). Hematoxylin/eosin staining, Masson’s trichrome staining, and immunohistochemical staining for HDAC3, CAIX, and Snail. Representative images (top) and quantification (bottom) of the staining are shown. Magnification, 200×. **b** The lung quantity (lung weight/body weight) was determined in the different groups. **c** The hydroxyproline content (μg/lung) in mouse lungs was determined in the different groups. **d** Immunoblotting using lysates from the lungs of control, bleomycin+siNC-injected, and bleomycin+siHDAC3-injected mice. Quantitative analysis of the immunoblotting results (right). N, normoxia; H, hypoxia; HD, histone deacetylase; CTL, control; BLM, bleomycin; CAIX, carbonic anhydrase IX; FN, Fibronectin. *P < 0.05, **P < 0.01, ****P < 0.0001 vs. control; ^#^*P* < 0.05, ^###^*P* < 0.001, ^####^*P* < 0^.^0001 vs. BLM + siNC.

The mouse model of bleomycin-induced pulmonary fibrosis is the most commonly used model to study human lung fibrosis. To examine whether inhibition of HDAC3 is effective in regulating lung fibrosis, we established a mouse pulmonary fibrosis model in 7-week-old male mice by intratracheal instillation of bleomycin. After injection of bleomycin, additional intratracheal injection of *HDAC3* siRNA decreased lung injury compared to that in mice with bleomycin injection alone, as demonstrated by hematoxylin/eosin and Masson’s trichrome staining (Fig. 3a). In addition, the expression levels of HDAC3, CAIX, and Snail were increased by 105-228%, 89-173%, and 41-828%, respectively, in lung tissues from mice with bleomycin-induced fibrosis, whereas mice with additional injection of *HDAC3* siRNA showed decreases of 32-90%, 53-98%, and 39-100% in the expression levels of HDAC3, CAIX, and Snail, respectively. Instillation of bleomycin increases lung weight because of the increase in inflammation; bleomycin also induces severe pulmonary fibrosis with an increase in hydroxyproline content in lung tissue^24,25^. Therefore, we measured the lung weight and hydroxyproline content. As shown in Fig. 3b, c, bleomycin treatment increased the quantity of pulmonary tissue and the pulmonary hydroxyproline content, whereas additional intratracheal instillation of *HDAC3* siRNA decreased the lung weight and hydroxyproline content compared to those in bleomycin-injected mice. Additionally, we examined the expression levels of EMT-related genes using lung tissues from control, bleomycin+siNC, and bleomycin+siHDAC3-injected mice. Bleomycin-induced Fibronectin, Snail, HIF-1α, HDAC3, *Ccn2* (Cellular communication network factor 2), *Serpine1* (Serpin family E member 1), and *Col1a1* were downregulated by additional *HDAC3* siRNA instillation (Fig. 3d and Supplementary Fig. 3). Collectively, these results indicate that inhibition of HDAC3 may suppress pulmonary fibrosis in mice through inhibition of EMT in the bleomycin-induced fibrosis model.

### HDAC3-miR-224 enhances alveolar EMT and fibroblast migration/invasion via inactivation of FOXA1 under hypoxia

**Fig. 4:**
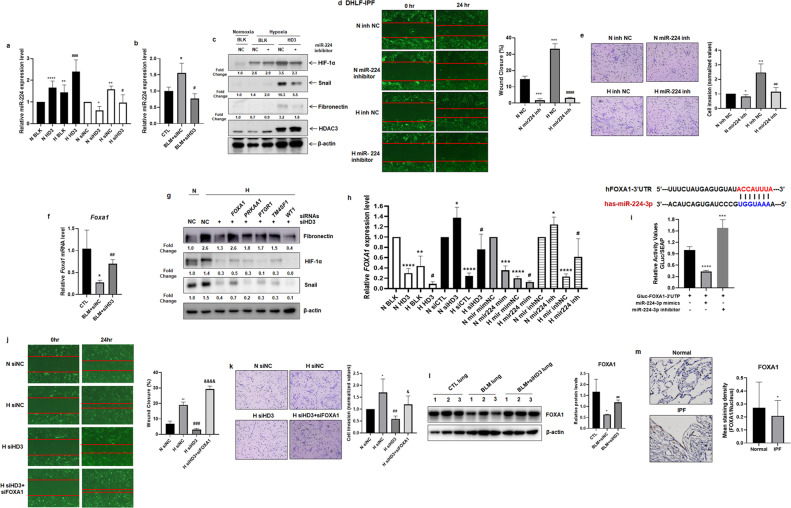
miR-224 and its target FOXA1 regulate the expression of EMT markers and the migration and invasion of DHLF-IPF cells under hypoxia. **a** qRT–PCR analysis of A549 cells overexpressing HDAC3 or A549 cells transfected with *HDAC3* siRNA and exposed to normoxia/hypoxia for 4 h. **b** qRT–PCR analysis of lung tissues from CTL/BLM + siNC-/BLM + siHDAC3-injected mice. **c** Immunoblot analysis of A549-HDAC3 cells treated with the miR-224 inhibitor and exposed to normoxia/hypoxia for 4 h. **d**, **e** DHLF-IPF cells were transfected with the miR-224 inhibitor and incubated under normoxia/hypoxia for 24–48 h. **d** Representative images showing migrated cells (left). Quantification of the percentage of healed area (right). Magnification, 40×. **e** Representative images showing the Matrigel invasion assay (left). Quantification of invaded cells (right). Magnification, 200×. **f** qRT–PCR analysis of lung tissues from CTL/BLM + siNC-/BLM + siHDAC3-injected mice. **g** Immunoblot analysis of A549 cells transfected with siRNAs against *HDAC3* and/or *FOXA1/PTGR1/PRKAA1/TM4SF1/WT1* and exposed to hypoxia for 4 h. **h** qRT–PCR analysis of A549-HDAC3 cells and A549 cells transfected with *HDAC3* siRNA, the miR-224 mimic, and the miR-224 inhibitor and exposed to normoxia/hypoxia for 24 h. **i** Sequence alignment of miR-224-3p and its target sites in the 3′ UTR of the human *FOXA1* gene (top). Luciferase reporter assay using A549 cells transfected with the miR-224 mimic/inhibitor and/or the *FOXA1*-3′UTR construct (bottom). **j**, **k** DHLF-IPF cells were transfected with siRNAs against *HDAC3* and *FOXA1* and incubated under normoxia/hypoxia for 24–48 h. **j** Quantification of the percentage of healed area (right). Magnification, 40×. **k** Representative images showing invaded cells (left). Quantification of invaded cells (right). Magnification, 200×. **l** Immunoblot analysis of lysates from the lungs of CTL/BLM + siNC-/BLM + siHDAC3-injected mice (left). Quantitative analysis of the immunoblotting results (right). **m** Immunohistochemical evaluation of FOXA1 in IPF patients’ lungs (n = 5). Representative images (left) and quantification (right) of staining. Magnification, 400×. BLM, bleomycin; IPF, idiopathic pulmonary fibrosis. *P < 0.05, **P < 0.01, ***P < 0.001, ****P < 0.0001 vs. normoxia or normal control; ^#^*P* < 0.05, ^##^*P* < 0.01, ^###^*P* < 0.001, ^####^*P* < 0.0001 vs. BLM + siNC or hypoxic control; ^&^*P* < 0.05, ^&&&&^*P* < 0.0001 vs. hypoxia+siHD3.

To identify the miRNAs that are regulated by HDAC3, we performed next-generation sequencing (NGS) analysis using HDAC3-overexpressing A549 cells. A number of miRNAs were elevated in hypoxic HDAC3-overexpressing cells. Among the miRNAs elevated in hypoxic HDAC3-overexpressing cells, we chose miR-224, based on a report that miR-224 is involved in driving EMT in malignant melanoma cell lines^15^. We found that miR-224 expression showed increases of 20-110% and 27.3-209% in normoxic and hypoxic HDAC3-overexpressing cells, respectively. However, miR-224 expression was decreased by 18-49% and 20.1-64.4% in normoxia- and hypoxia-exposed *HDAC3* siRNA-transfected cells, respectively (Fig. 4a). In addition, we confirmed that miR-224 expression was increased by 72.5-116% in lung tissues from bleomycin-injected mice and that it was decreased by 33.8-68.6% in lungs from bleomycin+siHDAC3-injected mice (Fig. 4b). We also examined whether miR-224 regulates HIF-1α, Snail, and Fibronectin in association with HDAC3 under hypoxic conditions. Treatment with the miR-224 inhibitor suppressed HDAC3-induced HIF-1α (34.3%), Snail (46.6%), and Fibronectin (44%) expression under hypoxic conditions, suggesting that miR-224 modulates HDAC3-induced HIF-1α, Snail, and Fibronectin expression under hypoxic conditions (Fig. 4c). However, the miR-224 inhibitor did not completely block HDAC3-induced HIF-1α, Snail, and Fibronectin expression, which means that there are other miRNAs that are regulated by HDAC3. Furthermore, the miR-224 inhibitor diminished the migration and invasion of DHLF-IPF cells, whereas the miR-224 mimic enhanced the migratory and invasive properties of DHLF-IPF cells under hypoxic conditions (Fig. 4d, e and Supplementary Fig. 4a, b). Therefore, we suggest that miR-224 induced by HDAC3 increases the expression of Snail and Fibronectin in alveolar epithelial cells and promotes the migration and invasion of fibroblast cells under hypoxic conditions.

We predicted the target genes of miR-224 using the TargetScan database and selected genes from this set that are associated with EMT for further study: Forkhead Box A1 (FOXA1), Prostaglandin Reductase 1 (PTGR1), Protein Kinase AMP-Activated Catalytic Subunit Alpha 1 (PRKAA1), Transmembrane 4 S Six Family Member 1 (TM4SF1), and Wilms tumor 1 (WT1). Among these genes, the mRNA levels of *FOXA1* and *TM4SF1* were decreased by 57.1-84.3% and 34.1-73%, respectively, in the lungs of bleomycin-injected mice in relation to the control group. On the other hand, in bleomycin+siHDAC3-injected mice lungs, the mRNA levels of *Foxa1* and *Tm4sf1* were higher by 87.9-233% and 35.2-176%, respectively, compared to those in the bleomycin-injected mice group (Fig. 4f and Supplementary Fig. 5). However, in alveolar epithelial cells, the protein levels of Fibronectin, HIF-1α, and Snail, which were reduced by HDAC3 depletion, were significantly restored by FOXA1 inhibition under hypoxic conditions (Fig. 4g). Therefore, we selected FOXA1 as a target gene of miR-224 in the regulation of hypoxia-induced EMT. The decrease in the *FOXA1* mRNA level induced by hypoxia exposure was suppressed by HDAC3 overexpression and miR-224 mimic treatment, whereas this change in *FOXA1* mRNA expression was reversed in *HDAC3* siRNA- and miR-224 inhibitor-transfected A549 cells (Fig. 4h). We predicted that the 3′UTR of *FOXA1* contains miR-224-binding sites using the TargetScanHuman database and validated their direct interaction using a luciferase reporter assay. The luciferase reporter assay revealed that FOXA1 was downregulated via direct binding of miR-224 to the 3′UTR of *FOXA1* (Fig. 4i). Therefore, we suggest that FOXA1 functions as a target gene of miR-224 during HDAC3-mediated EMT. It has been reported that FOXA1 plays an important role as an antagonist of EMT during pancreatic adenocarcinoma progression^26^. Moreover, FOXA1 functions as a negative regulator in the initial stages of lung cancer metastasis^27^. Therefore, we examined whether FOXA1 regulates the invasion and migration of fibroblast cells via a mechanism related to HDAC3 under hypoxia. The migration and invasion of DHLF-IPF cells, which were suppressed by HDAC3 inhibition, were restored—with increases of 608-1230% and 34.3-312%, respectively—after transfection with *HDAC3* and *FOXA1* siRNAs under hypoxia (Fig. 4j and k). In addition, the reduction in the FOXA1 protein level in the lungs of bleomycin-injected mice was ameliorated—increasing by 66.2-110%—in bleomycin+siHDAC3-injected mice (Fig. 4l). Finally, we examined the expression pattern of FOXA1 in lung tissues from human IPF patients using immunohistochemistry and observed that the FOXA1 expression level was reduced by 39.1-98.3% in IPF patient lungs (Fig. 4m). Taken together, these results suggest that HDAC3 induces miR-224 expression to drive alveolar EMT through FOXA1 downregulation under hypoxic conditions, which may accelerate pulmonary fibrosis.

## Discussion

Recently, studies have reported that HDAC3 promotes pulmonary fibrosis; Chen et al. reported that inhibition of HDAC3 and Nuclear Factor Erythroid-Derived 2-Related Factor-2 (Nrf2) mitigates pulmonary fibrosis^28^, and Zheng et al. suggested that HDAC3 accelerates pulmonary fibrosis by promoting EMT and inflammation through the Notch1 or STAT1 signaling pathway^29^. Although these findings are similar to those in our study in that all indicate that HDAC3 acts as a profibrotic factor in pulmonary fibrosis, our study differs in that we focused on the finding that HDAC3 promotes pulmonary fibrosis by accelerating hypoxia-induced EMT.

EMT may be classified into three types, which occur during normal organogenesis, the wound healing process, and malignancy. Among these processes, during the wound repair process, myofibroblasts are differentiated into fibroblasts by Transforming Growth Factor beta or Platelet-Derived Growth Factor secreted by epithelial cells in the wounded tissue^30^. EMT is regulated by a complex signaling network. During the chronic inflammation process, TGF-β1, oxidative stress, and hypoxia activate a signaling cascade that leads to activation and stabilization of the transcription factor Snail, which is a well-known EMT activator ^31^.

Hypoxia is an aggravating factor of cell inflammation and stimulates angiogenesis and fibrogenesis^32^. Chronic hypoxia is increasingly reported to be an important determinant of fibrosis and carcinogenesis in several tissues^33,34^. Although the involvement of inflammation and hypoxia in fibrosis has been consistently reported, the role of hypoxia in the induction of profibrotic and fibrosis marker gene expression in pulmonary fibrosis is poorly characterized. Therefore, elucidating the molecular mechanism underlying the exacerbation of pulmonary fibrosis by focusing on hypoxia-induced EMT is important in expanding the field of fibrosis research.

It has been reported that Snail is a direct target of HIF-1α in hypoxia-induced EMT in several types of cells^35–37^. In our study, although HDAC3 significantly increased Snail expression under hypoxic conditions, inhibition of *HIF1A* by siRNA prevented HDAC3 from elevating Snail expression under hypoxia (Fig. 1i). Furthermore, HDAC3 increased the transcriptional activity of HIF-1α by promoting the binding of HIF-1α to the HRE. Collectively, these observations indicate that HDAC3 stabilizes HIF-1α by increasing the transcriptional activity of genes with HREs; thus, stabilized HIF-1α seems to induce the EMT process via upregulation of Snail under hypoxic conditions.

Mesenchymal cells, especially fibroblasts, are observed in fibrotic lesions and at sites of wound healing^38^. The migration of fibroblast cells is an initiating event for wound healing and tissue remodeling^38^. The persistent migration of fibroblast cells is conducive to the enlargement of fibrotic lesions, which causes extreme tissue remodeling, finally resulting in a fibrotic scar^38^. Therefore, fibroblast migration is considered a critical property contributing to lung fibrosis. In our study, the HDAC3-miR224-FOXA1 axis effectively regulated the migration and invasion of fibroblast cells under hypoxia, suggesting that the HDAC3-miR-224-FOXA1 axis may be involved in pulmonary fibrosis by regulating the migratory and invasive properties of human fibroblast cells under hypoxic conditions.

To establish the mouse model of pulmonary fibrosis, we intratracheally administered bleomycin to 7-week-old male mice. Bleomycin-induced pulmonary fibrosis is the most commonly used model of human lung fibrosis despite its limitations with regard to explaining the progressive nature of human IPF. Since bleomycin induces inflammation in mice, leading to pulmonary fibrosis, it may not seem that this particular mouse model is related to a hypoxic environment and the EMT process. However, we found that the expression of the hypoxia marker CAXI as well as that of various mesenchymal markers was increased in lung tissues of bleomycin-treated mice. Therefore, it can be stated that a hypoxic environment is formed and EMT occurs in the setting of severe inflammation induced by bleomycin.

Given that several studies have used siRNAs against target genes to evaluate their effects on pulmonary fibrosis^39–41^, we intratracheally injected *HDAC3* siRNA into bleomycin-induced model mice to assess the effect of HDAC3 on pulmonary fibrosis. *HDAC3* siRNA administration alleviated bleomycin-induced pulmonary fibrosis in mice (Fig. 3).

Knoll et al. reported that E2F1 induces miR-224/452 expression to drive EMT through TXNIP downregulation in metastatic cells^15^. Furthermore, miR-224 is well known for its tumorigenic function in colorectal cancer^42,43^, clear cell renal cell carcinoma^44,45^, hepatocellular carcinoma^13^, and glioma^46^. However, the molecular mechanism underlying the role of miR-224 in pulmonary fibrosis is incompletely understood. Here, we suggest that miR-224 induced by HDAC3 increases the expression of EMT markers in alveolar epithelial cells; it also promotes the migration and invasion of fibroblast cells under hypoxic conditions (Fig. 4).

We predicted the target genes of miR-224 using the TargetScanHuman database and selected FOXA1 as a target gene of miR-224 in HDAC3-mediated alveolar EMT. FOXA1 acts as an important negative regulator of EMT during pancreatic adenocarcinoma progression through its positive regulation of E-cadherin and maintenance of the epithelial phenotype^26^. In our study, FOXA1 expression was suppressed in lung tissues of patients with pulmonary fibrosis and regulated the expression of EMT markers and the migration/invasion of fibroblast cells under hypoxia. In addition, miR-224 directly bound to the 3′UTR of *FOXA1*, leading to FOXA1 downregulation. Collectively, these findings indicate that miR-224 induced by HDAC3 functions as a profibrotic regulator through downregulation of FOXA1.

HDAC3 is a subtype of histone deacetylases, which removes acetyl groups from histone proteins or nonhistone proteins, leading to regulation of transcription factors. Therefore, we need to further study whether HDAC3 regulates the expression of miR-224 and FOXA1 through its enzymatic activity.

In conclusion, we demonstrated that HDAC3 promotes hypoxia-induced alveolar EMT through stabilization of HIF-1α via the AKT pathway and that it enhances the migration and invasion of fibroblasts. In addition, miR-224 functions as an important mediator of HDAC3-induced EMT via downregulation of FOXA1.

Although HDAC inhibitors are often considered promising therapeutic agents for fibrosis based on clinical trials, they have many side effects^47–49^. Therefore, the exact targets and mechanisms involved in the progression of pulmonary fibrosis must be further investigated. Thus, our study offers an effective molecular target for pulmonary fibrosis treatment by identifying the role of HDAC3 in alveolar EMT and fibroblast migration and invasion under hypoxic conditions.

## Supplementary information

Supplementary figures

## Data Availability

The datasets used and/or analyzed during the current study are available from the corresponding author on reasonable request.
