# Supplementary material for: Histone deacetylase 3 promotes alveolar epithelial–mesenchymal transition and fibroblast migration under hypoxic conditions
Source: Exp Mol Med. 2022 Jul 8;54(7):922–31. doi: 10.1038/s12276-022-00796-y (PMC9355949; doi:10.1038/s12276-022-00796-y)
Supplement: Supplementary file 1 — Supplementary figures [file 12276_2022_796_MOESM1_ESM.pdf]

Histone deacetylase 3 promotes alveolar epithelial–mesenchymal transition and fibroblast migration under hypoxic conditions

Sung Hwan Jeong[1,*], Eun Suk Son[2,*], Young Eun Lee[1], Sun Young Kyung[1],

Jeong-Woong Park[1,#], Se-Hee Kim[3,#]

[1]Department of Allergy, Pulmonary and Critical Care Medicine, Gachon University Gil Medical Center, Incheon, South Korea; [2]Department of Medicine, College of Medicine, Gachon University, Incheon, South Korea; [3]Gachon Medical Research Institute, Gachon University Gil Medical Center, Incheon, South Korea

Authors and affiliations

Se-Hee Kim, Gachon Medical Research Institute, Gachon University Gil Medical Center, Incheon, South Korea. sehee0423@gilhospital.com

Jeong-Woong Park, Department of Allergy, Pulmonary and Critical Care Medicine, Gachon University Gil Medical Center, Incheon, South Korea. jwpark@gilhospital.com

Sung Hwan Jeong, Department of Allergy, Pulmonary and Critical Care Medicine, Gachon University Gil Medical Center, Incheon, South Korea. jsw@gilhospital.com

Eun Suk Son, Department of Medicine, College of Medicine, Gachon University, Incheon, South Korea. eunsuk0607@hanmail.net

Young Eun Lee, Department of Allergy, Pulmonary and Critical Care Medicine, Gachon University Gil Medical Center, Incheon, South Korea. lye0610@naver.com

Sun Young Kyung, Department of Allergy, Pulmonary and Critical Care Medicine, Gachon University Gil Medical Center, Incheon, South Korea. light@gilhospital.com

[*]Contributed equally

[#] Correspondence: jwpark@gilhospital.com (J.-W.P.); sehee0423@gilhospital.com (S.-H.K.)

Correspondence to:

Jeong-Woong Park, MD, PhD., Department of Medicine, Gachon University Gil Medical Center, 38-13, Dokjeom-ro 3 beon-gil, Namdong-gu, Incheon 21565, Republic of Korea.
Telephone: +82-32-460-8416; Fax: +82-32-469-4320
E-mail: jwpark@gilhospital.com

Se-Hee Kim, PhD., Gachon Medical Research Institute, Gachon University Gil Medical Center, 38-13, Dokjeom-ro 3 beon-gil, Namdong-gu, Incheon 21565, Republic of Korea.
Telephone: +82-32-458-2872
E-mail: sehee0423@gilhospital.com

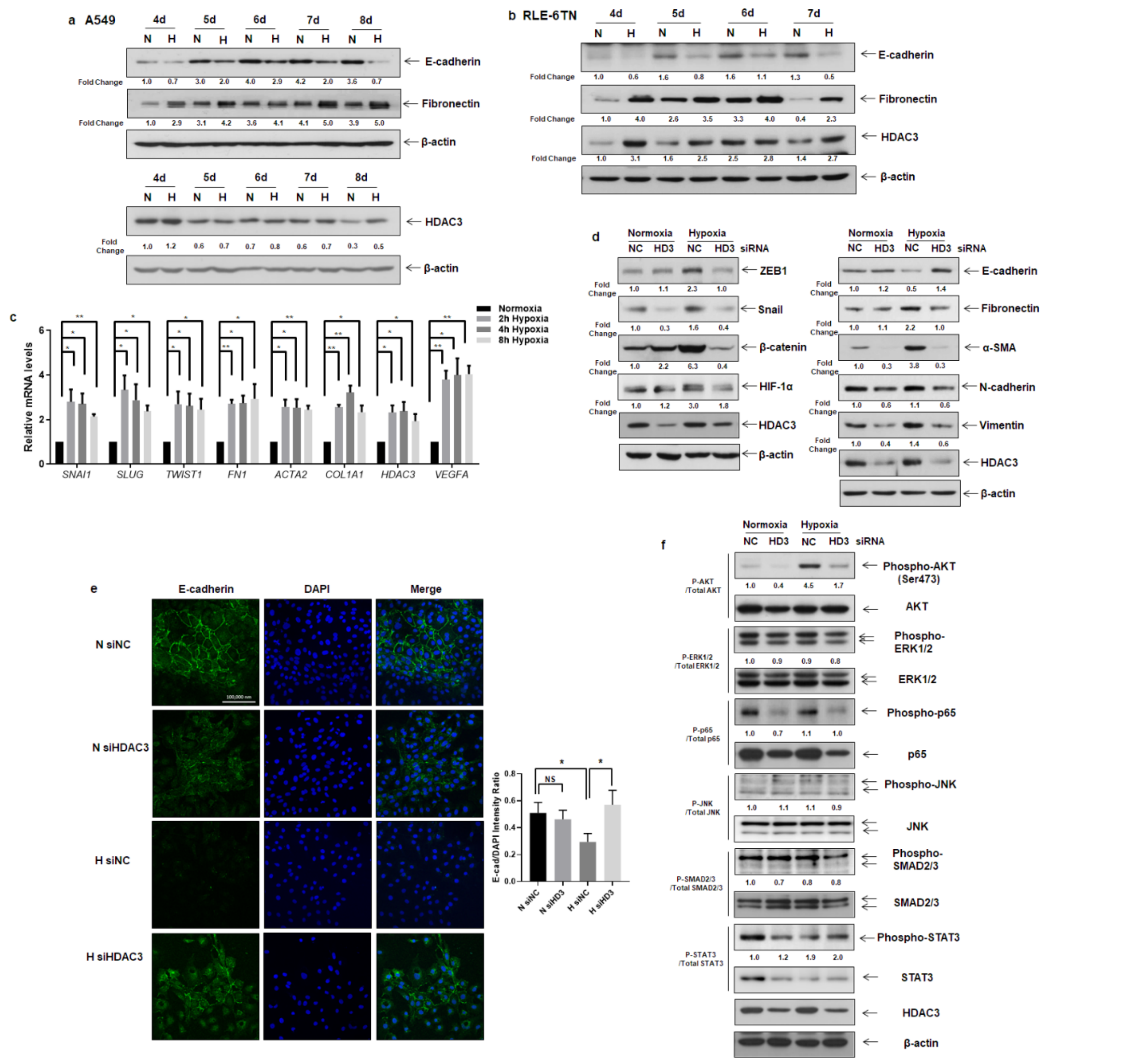

Supplementary Fig. 1. HDAC3 is effective in the regulation of EMT markers under hypoxia. **a** and **b** Human alveolar epithelial A549 cells (**a**) and rat alveolar epithelial RLE-6TN cells (**b**) were exposed to normoxia (21% O_2) or hypoxia (1% O_2) for 4–8 days. Immunoblotting for detection of E-cadherin, Fibronectin, and HDAC3 proteins was performed. β-actin was used as a loading control. N, normoxia; H, hypoxia. **c** Quantitative reverse-transcription (qRT)-PCR analysis of EMT-related genes in A549 cells exposed to normoxia/hypoxia for 2–4 h. SNAI1, snail1; FN, fibronectin; ACTA2, actin alpha2, smooth muscle; COL1A1, collagen type I alpha 1 chain; HDAC, histone deacetylase; VEGFA, vascular endothelial growth factor A. $^{*}P < 0.05$ and $^{**}P < 0.01$ vs. normoxia control; paired *t*-test. **d** RLE-6TN cells were transfected with siRNA for *HDAC3* and exposed to normoxia or hypoxia for 4 h or 4 days. Immunoblotting for detection of ZEB1, Snail, β-catenin, HIF-1α, HDAC3, E-cadherin, Fibronectin, α-SMA, N-cadherin, and Vimentin proteins was performed. β-actin was used as a loading control. NC, negative control; HD, histone deacetylase; ZEB1, zinc finger E-box binding homeobox 1. **e** RLE-6TN cells were transfected with siRNA for *HDAC3* and exposed to normoxia or hypoxia for 4 days. Cells grown on cover slips were fixed and stained with E-cadherin antibody. E-cadherin and DAPI were visualized using confocal fluorescence microscopy. Quantification of

E-cadherin-positive cells (right). N, normoxia; H, hypoxia; CTL, control; HD, histone deacetylase; NS, not significant. $^{*}P < 0.05$ vs. normoxia or hypoxia control; paired *t*-test. Scale bar, 100 µm. **f** RLE-6TN cells were transfected with *HDAC3* siRNA and exposed to normoxia or hypoxia for 4 h. Cells were lysed, and proteins were subjected to sodium dodecyl sulfate polyacrylamide gel electrophoresis, followed by Western blot analysis for the detection of phospho-AKT, AKT, phospho-ERK1/2, ERK1/2, phospho-p65, p65, phospho-JNK, JNK, phospho-SMAD2/3, SMAD2/3, phospho-STAT3, STAT3, and HDAC3 proteins. β-actin was used as a loading control. N, normoxia; H, hypoxia; CTL, control; NC, negative control; HD, histone deacetylase; AKT, Ak strain transforming; ERK, extracellular signal regulated kinase; JNK, c-jun N-terminal kinase; SMAD, small-mothers-against-decapentaplegic; STAT, signal transducer and activator of transcription.

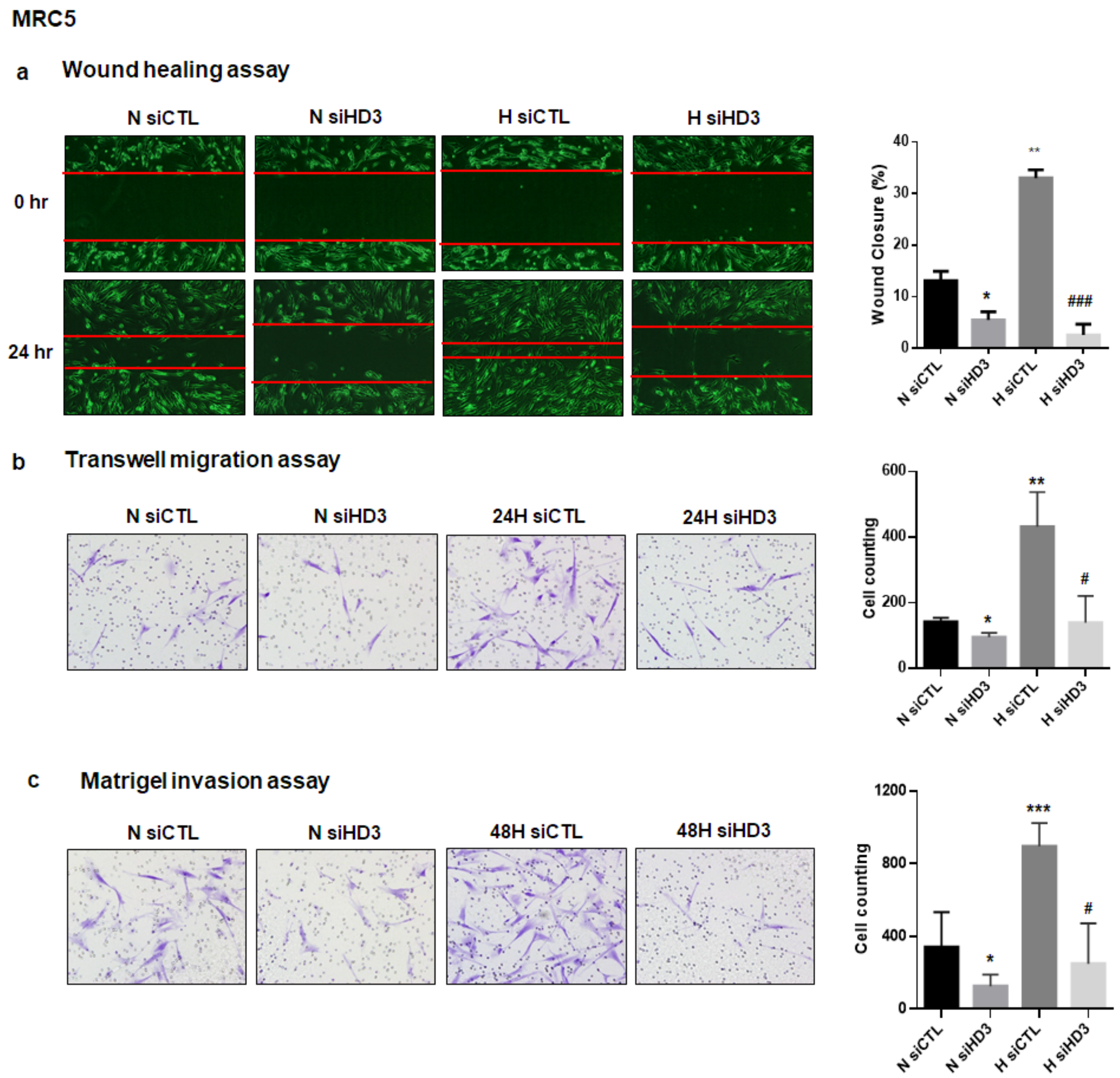

Supplementary Fig. 2. Inhibition of HDAC3 prevents the migration and invasion of fibroblast cells under hypoxic conditions. **a** MRC5 cells were transfected with *HDAC3* siRNA (#1 and #2) and were scratched and then incubated under normoxia or hypoxia for 24 h. Representative images showing wound healing assay (left). Quantification of percentage of recovery area (right). Magnification, 40×. N, normoxia; H, hypoxia; CTL, control; HD, histone deacetylase. $^{*}P < 0.05$ and $^{**}P < 0.01$ vs. normoxia control, $^{\#\#\#}P < 0.001$ vs. hypoxia control; unpaired *t*-test. **b** Negative control or *HDAC3* siRNAs-transfected MRC5 cells were seeded into transwells and exposed to normoxia/hypoxia for 24 h. Cells on the upper surface of the transwells were stained and counted. Representative images showing transwell migration assay (left). Quantification of migratory cells (right). Magnification, 200×. N, normoxia; H, hypoxia; CTL, control; HD, histone deacetylase. $^{*}P < 0.05$ and $^{**}P < 0.01$ vs. normoxia control, $^{\#}P < 0.05$ vs. hypoxia control; unpaired *t*-test. **c** Negative control or *HDAC3* siRNAs-transfected MRC5 cells were seeded into transwells coated with matrigel and exposed to normoxia/hypoxia for 48 h. Cells on the upper surface of the transwells were stained and counted. Representative images showing matrigel invasion assay (left). Quantification of invasive cells (right). Magnification, 200×. N, normoxia; H, hypoxia; CTL, control; HD, histone deacetylase. $^{*}P < 0.05$ and $^{***}P < 0.001$ vs. normoxia control, $^{\#}P < 0.05$ vs. hypoxia control; unpaired *t*-test.

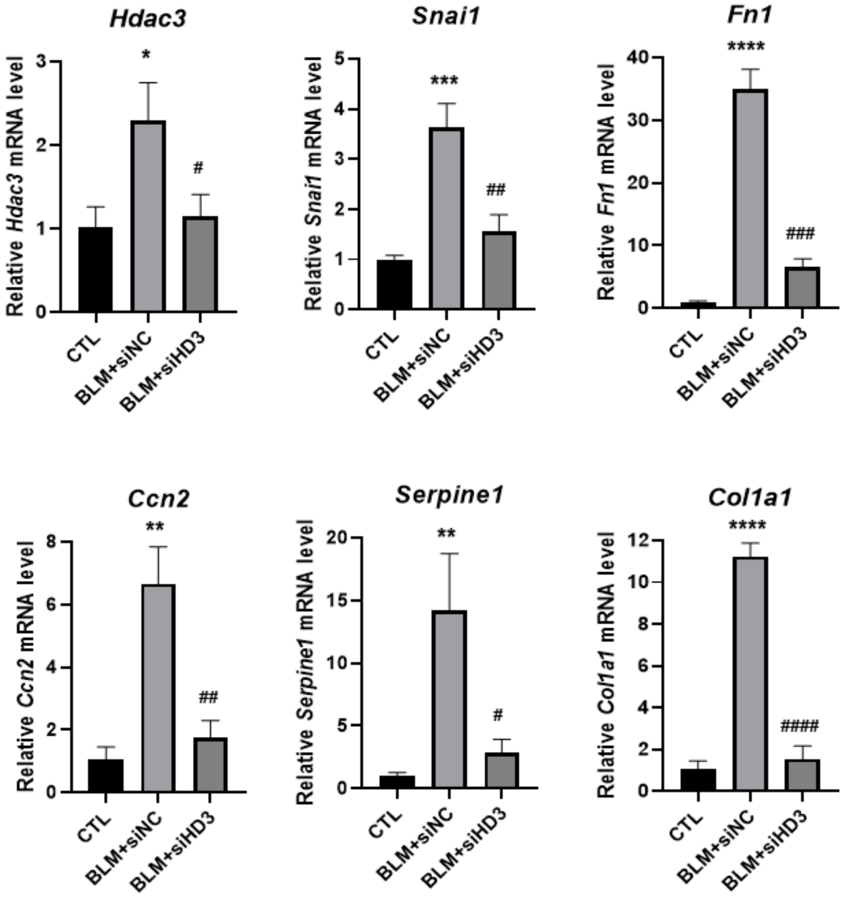

Supplementary Fig. 3. Inhibition of HDAC3 represses mRNA levels of mesenchymal markers in mice lungs. QRT-PCR analysis of EMT related genes (*Snai1*, *Fn1*, *Ccn2*, *Serpine1*, and *Col1a1*) and *Hdac3* in lungs from control, bleomycin+siNC, bleomycin+siHD3 mice. CTL, control; BLM, bleomycin; NC, negative control; HD, HDAC; Fn, fibronectin; Ccn2, cellular communication network factor 2; Serpine1, serpin family E member 1; Col1a1, collagen type I α1. $^{*}P < 0.05$, $^{**}P < 0.01$, $^{***}P < 0.001$, $^{****}P < 0.0001$ vs. control, $^{\#}P < 0.05$, $^{\#\#}P < 0.01$, $^{\#\#\#}P < 0.001$, $^{\#\#\#\#}P < 0.0001$ vs. BLM+siNC; unpaired *t*-test.

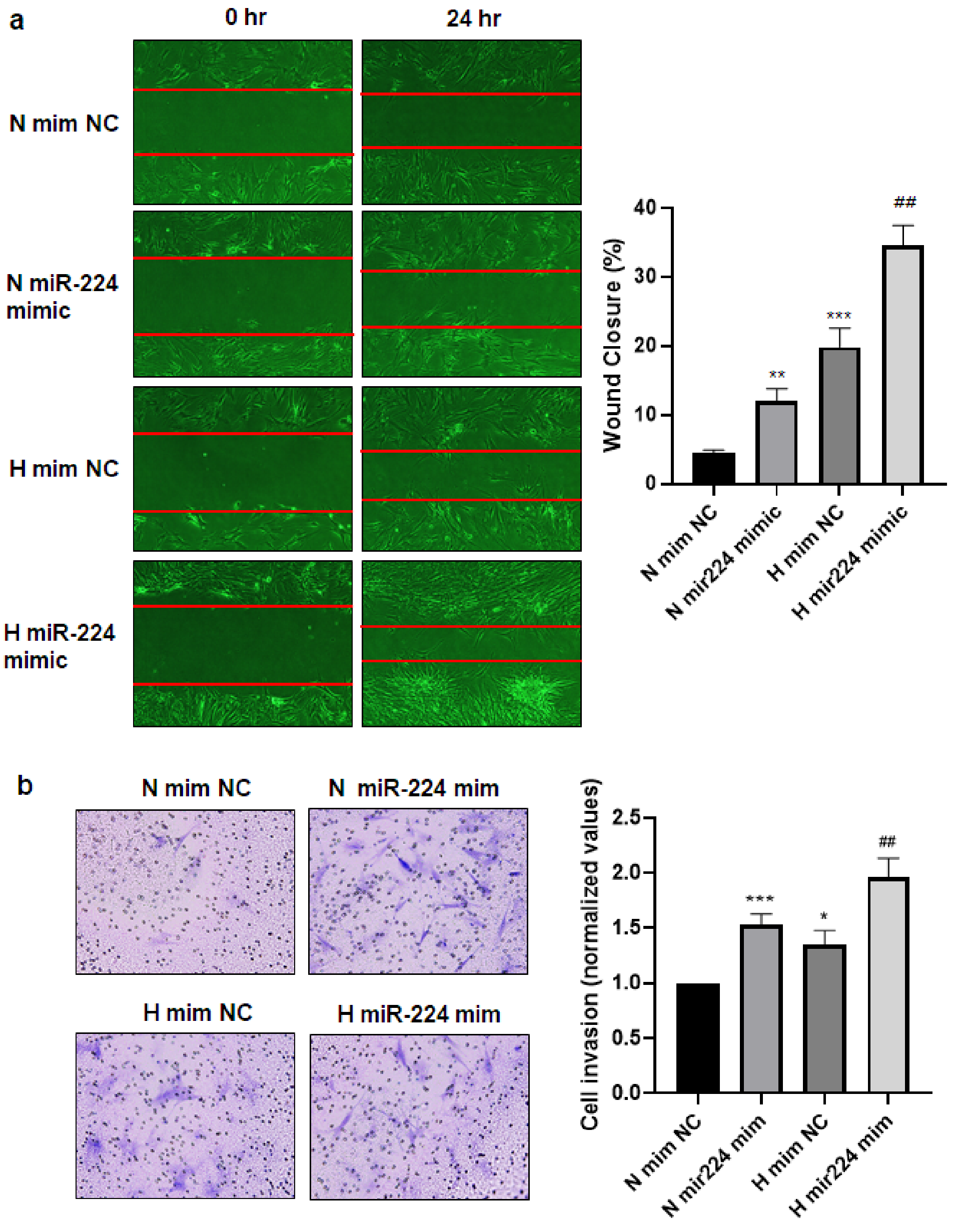

Supplementary Fig. 4. miR-224 mimic promotes the migration and invasion of DHLF-IPF cells in hypoxic conditions. (**a** and **b**) DHLF-IPF cells were transfected with miR-224 mimic and incubated under normoxia/hypoxia for 24–48 h. **a** Representative images showing wound healing assay (left). Quantification of percentage of recovery area (right). Magnification, 40×. **b** Representative images showing Matrigel invasion assay (left). Quantification of invasive cells (right). Magnification, 200×. N, normoxia; H, hypoxia; mim, mimic. $^{*}P < 0.05$, $^{**}P < 0.01$, and $^{***}P < 0.001$ vs. normoxia control, $^{\#\#}P < 0.01$ vs. hypoxia control; unpaired *t*-test.

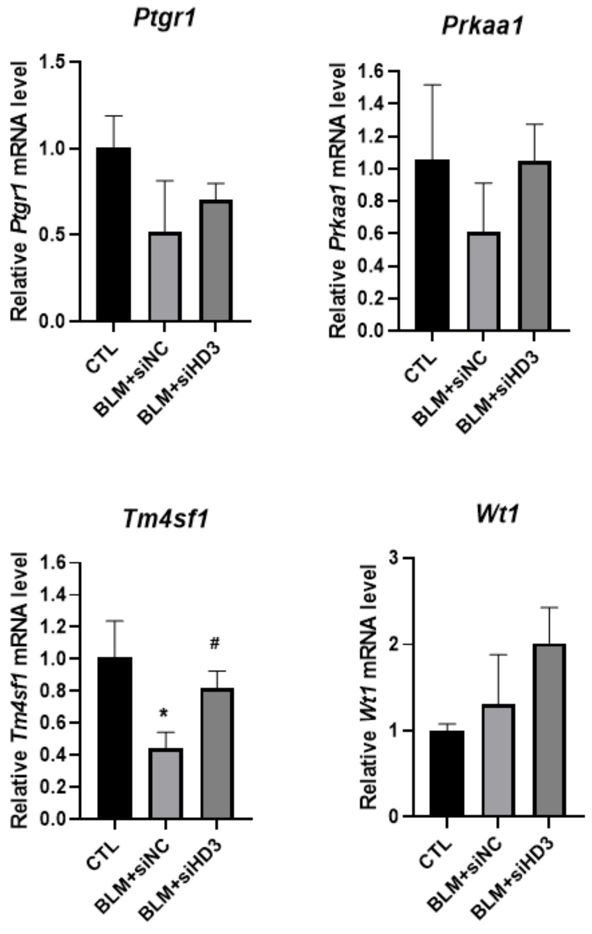

Supplementary Fig. 5. Expression of *Ptgr1*, *Prkaa1*, *Tm4sf1*, and *Wt1* in mice lungs. QRT-PCR analysis of *Ptgr1*, *Prkaa1*, *Tm4sf1*, and *Wt1* in lungs from control, bleomycin+siNC, bleomycin+siHD3 mice. CTL, control; BLM, bleomycin; NC, negative control; HD, HDAC; Ptgr1, Prostaglandin Reductase 1; Prkaa1, Protein Kinase AMP-Activated Catalytic Subunit Alpha 1; Tm4sf1, Transmembrane 4 S Six Family Member 1, and Wt1, Wilms tumor 1. $^{*}P < 0.05$ vs. control, $^{\#}P < 0.05$ vs. BLM+siNC; unpaired *t*-test.

Supplemental Methods

Cell lines and hypoxic condition

Rat alveolar epithelial cell line, RLE-6TN and MRC-5 cells were obtained from the ATCC (Manassas, VA, USA) and the Korean Cell Line Bank (Seoul, South Korea), respectively. RLE-6TN cells were cultured in Ham's F12 medium with 2 mM L-glutamine (Gibco Cell Culture, Carlsbad, CA, USA) containing 10% fetal bovine serum (FBS)(Gibco) and 1% penicillin-streptomycin (PS)(Gibco), and MRC-5 cells were grown in DMEM (Gibco Cell Culture) supplemented 10% FBS and 1% PS. For hypoxic incubation, cells were incubated in a hypoxic incubator (New Brunswick Scientific, Edison, NJ, USA) with a humidified environment consisting of 1% O_2, 5% CO_2, and 94% N_2.

RNA interference, miR-224 inhibitors, miR-224 mimics and transfection

Cells were transfected with 50 nM siRNAs (Bioneer, Daejeon, South Korea) or 50 nM miR mimic (Bioneer) using Lipofectamine 2000 (Invitrogen, Carlsbad, CA, USA) according to the manufacturer's protocols.

The sequences for siRNAs and miR-224 inhibitors were as follows: *HDAC3 #1* (5'-AAU CAG AAC UCA CGC CAG UAU-3'), *HDAC3 #2* (5'- GAU GCU GAA CCA UGC ACC-3'), miR-224-3p inhibitor (5'-AAA AUG GUG CCC UAG UGA CUA CA-3'), and miR-224-mimic (5'-UCA AGU CAC UAG UGG UUC CGU UUA G-3').

Western blot analysis

For protein preparation, cells were lysed with radioimmunoprecipitation assay buffer (50 mM Tris-HCl pH 8.0, 150 mM NaCl, 0.5% sodium deoxycholate, 0.1% sodium dodecyl

sulfate, and 1% NP-40) or whole cell lysate buffer (10 mM 4-(2-hydroxyethyl)-1-piperazineethanesultonic acid pH 7.9, 400 mM NaCl, 0.1 mM ethylenediaminetetraacetic acid, 5% glycerol, 1 mM dithiothreitol). Primary antibodies to Fibronectin (Santa Cruz Biotechnology, Santa Cruz, CA, USA), E-cadherin (Abcam, Cambridge, UK), α-SMA (Abcam), Zinc finger E-box binding homeobox 1 (ZEB1) (Cell Signaling), N-cadherin (Cell Signaling), Vimentin (Cell Signaling), β-catenin (Cell Signaling), Hypoxia-inducible factor 1- alpha (HIF-1α) (Novus, Centennial, CO, USA), Snail (Cell Signaling), HDAC3 (Santa Cruz Biotechnology), phosphor-Ak strain transforming (AKT) (Cell Signaling), AKT (Cell Signaling), phospho-Extracellular signal regulated kinase (ERK)1/2 (Cell Signaling), ERK1/2 (Cell Signaling), phospho-p65 (Cell Signaling), p65 (Cell Signaling), phospho-c-jun N-terminal kinase (JNK) (Cell Signaling), JNK (Cell Signaling), phospho-small-mothers-against-decapentaplegic (SMAD)2/3 (Cell Signaling), SMAD2/3 (Cell Signaling), phospho-signal transducer and activator of transcription (STAT)3 (Cell Signaling), STAT3 (Cell Signaling), and β-actin (Santa Cruz Biotechnology) were used. The samples were normalized to β-actin or a ratio of phosphorylated protein to total protein using ImageJ software.

Quantitative reverse transcription PCR (qRT-PCR)

Total RNA was prepared from cells using RNAiso Plus reagent (Takara Bio), and the PrimeScript First Strand cDNA Synthesis Kit (Takara Bio) was employed for cDNA transcription. qRT-PCR was performed using SYBR Green I Universal PCR Master Mix (Takara Bio) on a CFX 96 real-time system (Bio-Rad). Samples were loaded in triplicate and the experiment was repeated more than three times. The fold change of target gene expression relative to the Glyceraldehyde 3-phosphate dehydrogenase (*GAPDH*)

endogenous control gene was determined following the manufacturer's instructions. The primer sequences for PCR reactions were as follows: human *SNAI1*, 5'-CCC CAA TCG GAA GCC TAA CT-3' (forward) and 5'-GCT GGA AGG TAA ACT CTG GAT TAG A-3' (reverse); human *SLUG*, 5'-ACG CCC AGC TAC CCA ATG-3' (forward) and 5'-CGC CCC AAA GAT GAG GAG TA-3' (reverse); human *TWIST1*, 5'-GCG CTG CGG AAG ATC ATC-3' (forward) and 5'-GGT CTG AAT CTT GCT CAG CTT GT-3' (reverse); human *FN1* (Fibronectin 1), 5'- CCA TCG CAA ACC GCT GCC AT -3' (forward) and 5'-AAC ACT TCT CAG CTA TGG GCT T-3' (reverse); human *ACTA2* (actin alpha 2, smooth muscle), 5'-ACT GAG CGT GGC TAT TCC TTC GTT-3' (forward) and 5'-GCA GTG GCC ATC TCA TTT TCA-3' (reverse); human *COL1A1* (collagen, type I, alpha 1), 5'-TCG GCG AGA GCA TGA CCG AT-3' (forward) and 5'-GGC CAC GCT GTT CTT GCA GT-3' (reverse); human *HDAC3* (histone deacetylase 3), 5'- TAG ACA AGG ACT GAG ATT GCC-3' (forward) and 5'-GTG TTA GGG AGC CAG AGC C-3' (reverse); human *VEGFA* (vascular endothelial growth factor A), 5'-ATC TTC AAG CCA TCC TGT GTG C-3' (forward) and 5'-CAA GGC CCA CAG GGA TTT TC-3' (reverse); human *GAPDH*, 5'-AGG TCG GAG TCA ACG GAT TTG G-3' (forward) and 5'-ACA GTC TTC TGG GTG GCA GTG ATG-3' (reverse); mouse *Hdac3*, 5'-CAC CCG CAT CGA GAA TCA GA-3' (forward) and 5'-CTT CCC ACC ACA GAG GTG AC-3' (reverse); mouse *Snai1*, 5'- CCA CTG CAA CCG TGC TTT T-3' (forward) and 5'- CAC ATC CGA GTG GGT TTG G-3' (reverse); mouse *Fn1*, 5'- CCG TGG GAT GTT TTG AGA C-3' (forward) and 5'- GGC AAA AGA AAG CAG AGG-3' (reverse); mouse *Ccn2* (cellular communication network factor 2), 5'- GTG TGT GAC GAG CCC AAG GA-3' (forward) and 5'- CAG TTG GCT CGC ATC ATA GTT G-3' (reverse); mouse *Serpine1* (serpin family E member 1), 5'-AGT CTT TCC GAC CAA GAG CA-3' (forward) and 5'- ATC ACT TGC CCC ATG AAG AG-3' (reverse); mouse *Col1a1*, 5'- CAC CCT CAA GAG CCT GAG TC -3' (forward) and 5'- GCT TCT TTT CCT TGG GGT TC-3' (reverse); mouse *Ptgr1*

(prostaglandin reductase 1), 5'-GAC AAA GCT GCC TGT AGA GTG G-3' (forward) and 5'-GCT GAC CAT CAC GGT TTC TCC A-3' (reverse); mouse *Prkaa1* (protein kinase AMP-activated catalytic subunit alpha 1), 5'- GGT GTA CGG AAG GCA AAA TGG C-3' (forward) and 5'- CAG GAT TCT TCC TTC GTA CAC GC-3' (reverse); mouse *Tm4sf1* (transmembrane 4 S six family member 1), 5'- GGA TGA AGA GGA CTG CTG TGG T-3' (forward) and 5'- CCA CGA TTC CAA TCA GAG CAG C-3' (reverse); mouse *Wt1* (Wilms tumor 1), 5'-GGT TTT CTC GCT CAG ACC AGC T-3' (forward) and 5'- ATG AGT CCT GGT GTG GGT CTT C-3' (reverse); mouse *Gapdh*, 5'-TGT GTC CGT CGT GGA TCT GA-3' (forward) and 5'-CCT GCT TCA CCA CCT TCT TGA-3' (reverse).

Immunocytochemistry

Cells were seeded on coverslips coated with 0.1% gelatin in 6-well plates and incubated in hypoxic/normoxic conditions for 4 days, and then fixed in 3.7% paraformaldehyde. After permeabilizing in 0.5% Triton X-100/phosphate-buffered saline (PBS) and several washings with PBS, cells were incubated with E-cadherin antibody (Abcam) diluted in 1% bovine serum albumin (BSA)/PBS-T for 1 h at room temperature. Afterwards, cells were incubated with AlexaFluor 488 donkey anti-rabbit IgG antibody (Life Technologies, Carlsbad, CA, USA) and mounted in Vectashield containing DAPI (Vector Laboratories, Burlingame, CA, USA). Images were taken at 200 x magnification with a LSM710 confocal microscope (Carl Zeiss, Jena, Germany).

Wound healing assay

siRNAs or miR-224 mimic-transfected cells were seeded in 60-mm dishes and then scratched using pipette tips after 24 h of incubation. Images were taken at 0 and 24 h

using an Olympus CFX41 microscope (Hamburg, Germany). The cell migration area was quantified using ImageJ software and calculated as described previously[1].

Transwell migration assay

The experimental process was described previously[2]. Briefly, siRNAs-transfected cells were seeded into transwells with the lower surface coated with 0.2% gelatin and 20 % FBS-containing medium was used as a chemoattractant. After 24 h of incubation under normoxic or hypoxic conditions, the cells on the lower membrane were fixed and stained using crystal violet (YD Diagnostics, Gyeonggi, South Korea) to count. The experiments were independently performed in triplicate.

Matrigel invasion assay

This assay was done as previously described[2]. Briefly, siRNAs or miR-224 mimic-transfected cells were seeded into transwells with the lower surface coated with 0.2% gelatin and the upper surface coated with Matrigel (BD Biosciences, San Jose, CA, USA). After 48 h of incubation under normoxic or hypoxic conditions, the cells on the lower membrane were fixed and stained using crystal violet (YD Diagnostics) to count. The experiments were independently performed in triplicate.

Statistics

Results were analyzed using GraphPad Prism (San Diego, CA, USA). Statistical significance was assessed using the Mann–Whitney test and two-tailed paired *t*-test; $p < 0.05$ was considered significant.

Supplemental references

1 Kim, J. H. et al. Dexamethasone inhibits hypoxia-induced epithelial-mesenchymal transition in colon cancer. *World J. Gastroenterol.* **21**, 9887-9899 (2015).

2 Son, E. S. et al. Coix lacryma-jobi var. ma-yuen Stapf sprout extract has anti-metastatic activity in colon cancer cells in vitro. *BMC Complem. Altern. Med.* **17**, 486 (2017).
